# Adverse competition-related cognitions and it’s relation to satisfaction and subjective performance: a validation study in a sample of English-speaking athletes

**DOI:** 10.1038/s41598-025-16077-3

**Published:** 2025-08-25

**Authors:** Alena Michel-Kröhler, Michèle Wessa, Stefan Berti

**Affiliations:** 1 Department of Clinical Psychology and Neuropsychology, Institute for Psychology, Johannes Gutenberg-University Mainz, Wallstrasse 3, 55122 Mainz, Germany; 2 Department of Neuropsychology and Psychological Resilience Research, Central Institute of Mental Health, Mannheim, Germany; 3 German Cancer Research Center (DKFZ), Division of Cancer Survivorship and Psychological Resilience, Heidelberg, Germany; 4 Leibniz Institute for Resilience Research (LIR), Mainz, Germany

**Keywords:** Performance psychology, Scale development, Competitive sports, Cognitive interference, Population-based differences, Human behaviour, Psychology

## Abstract

This study aimed at examining the reliability and validity of the Adverse Competition-related Cognitions Questionnaire (ACCQ) in an English-speaking sample of athletes. The ACCQ is a performance-focused measure that captures six different areas of adverse competition-related cognitions– athletic comparison, coach devaluation, devaluation of one’s own performance, appreciation by coach and family, inner resistance against competitions, and general exhaustion. Data from 278 athletes (*M*_age_ = 27.64, age range = 16–68 years) from different sports were collected and subjected to confirmatory factor analysis, which confirmed the 6-factor solution of the translated ACCQ (CFI = 0.915; RMSEA = 0.056). In addition, the different subscales of the ACCQ showed sufficient internal consistency (Cronbach’s alpha > 0.60). Furthermore, we examined the relationships with cognitive interference, satisfaction in three different domains (i.e., general in life, sporting development, and athletic performance) and athletes’ subjective performance evaluations (i.e., performance and peak performance in the previous season and confidence in achieving their goals in the upcoming season). Results indicated positive correlations with athletes’ cognitive interference (i.e., construct validity in terms of a nomological network), low negative relations with athletes’ satisfaction in the three different domains and with the three parameters of subjective performance evaluation (i.e., concurrent validity). Implications of these findings and perspectives for future research are discussed.

## Introduction

Competitions are a central element of high-performance and professional sports and often place high psychological demands on athletes. These demands can result from a number of stressors, such as internal, external and organizational ones^1^. In addition, athletes often face setbacks such as failure, injury or prolonged performance plateaus. Taken together, these factors can significantly increase an athlete’s vulnerability to mental health problems and, in some cases, contribute to the development of mental illness^2,3^.

Furthermore, athletes often attach great importance to competitions. In this context, athletes are constantly exposed to comparisons - either between their current performance and their personal goals or between themselves and their opponents. These comparisons can highlight discrepancies that are perceived as negative, generating additional psychological pressure^4,5^.As a result, negative thoughts may emerge that can influence the sporting behavior and subjective outcomes of a competition. Consequently, it is important to work with athletes to develop specific strategies for dealing with adverse thoughts and to support them in their optimal performance development. To specifically plan and implement such counseling for athletes, valid and reliable measures are needed that capture the extent to which adverse cognitions are prevalent in athletes. In addition, the use of such measures would allow an assessment of how these adverse cognitions might be related to different aspects of athletes’ sporting behavior or mental health.

So far there are a lot of questionnaires in English that capture self-talk (e.g., Organic Self-Talk Questionnaire for Sport; OSTQS^6^, Automatic Self-Talk Questionnaire for Sports; ASTQS^7^, cognitive interference (e.g., Thoughts Occurrence Questionnaires Sport; TOQS^8^, or irrational performance beliefs (irrational Performance Beliefs Inventory; iPBI^9^, but these questionnaires do not measure sport-specific adverse performance-limiting thoughts at their core. In contrast, a German questionnaire measuring adverse competition-related cognitions, the Adverse Competition-related Cognitions Questionnaire (ACCQ^10^,has recently been developed and appears suitable for measuring the prevalence of potential negative cognitions. Therefore, the aim of the study was to validate and test the ACCQ for the application within a sample of English-speaking athletes.

## Adverse competition-related cognitions

The ACCQ is a 26-item self-report measure that captures a series of thoughts that may occur during a competition. The ACCQ consists of six competition-related subscales: (1) ‘Athletic comparison’, which measures the tendency of athletes to compare themselves or their performance with others, (2) ‘Coach devaluation’ refers to thoughts related to the communication with the coach or the perceived competence of the coach, (3) ‘Devaluation of one’s own performance’ consists of thoughts concerning the retrieval of performance and the current state of the athlete, (4) ‘Appreciation by coach and family’ addresses the athlete’s expectations regarding the appreciation of his or her athletic performance by the coach or parents, (5) ‘Inner resistance against competitions’ describes different types of escaping thoughts, and lastly, (6) ‘General exhaustion’, indicates athletes’ feelings of exhaustion before or during a competition. Responses are rated on a 5-point scale ranging from ‘1’ (*never*) to ‘5’ (*always*) and are related to performance and performance-related aspects (e.g., self-esteem, self-efficacy, and choking under pressure). The ACCQ currently exists only in a German version. To be able to carry out cross-cultural comparisons in the future and to use the questionnaire with English-speaking athletes, the ACCQ must first be translated and validated.

### Cognitive interference, satisfaction ratings and subjective performance evaluation

We considered athletes’ cognitive interference (i.e., thoughts that interfere with concentration and prevent the athlete from focusing on the sporting task^11^, as an additional marker of the construct validity of the ACCQ, and satisfaction as well as the subjective performance evaluation as markers of concurrent validity of the ACCQ. Due to the partial proximity in content, we used the TOQS in accordance with the original validation study^10^ for further construct validation of our translated version of the ACCQ. Michel-Kröhler and colleagues^10^ reported low to medium correlations between the ACCQ subscales and the TOQS scales.

Regarding satisfaction, we captured athletes’ general satisfaction with life (which is based on the evaluation of one’s own life from a long-term perspective, and which is a cognitive component of subjective well-being^12^, and with their sporting development also according to Michel-Kröhler et al.^10^. Previous findings indicate that the higher the adverse competition-related cognitions the lower the athletes’ satisfaction in these two domains^10^. In addition, we examined satisfaction with athletic performance in the last season, as performance could be a strong source for the development of adverse competition-related cognitions that could influence future sport behavior. Lastly, we captured the athletes’ subjective performance evaluation with different parameters, as this enables a performance comparison between athletes competing in different sports or occupying distinct positions in the team^13^. Furthermore, in competitions, the perception of the situation or challenge in relation to one’s own abilities usually influences the (subjective) outcome of the performance^14^. This can, for example, cause an athlete to evaluate their performance and related information more negatively than the external perspective or objective outcome^15^. This is also in line with Martin and Tesser^5^, who emphasize that the objective fulfillment of a task does not always correspond to the subjective achievement of a goal. Therefore, we used three different parameters to measure the subjective performance evaluation of athletes from different sports in our sample: (1) the rating of overall performance, (2) the percentage of peak performance retrieval in the previous season, and (3) the confidence in achieving their goals in the upcoming season.

## Current study

The present study aimed at confirming the factorial structure of the ACCQ in an English-speaking sample of athletes. To further validate the construct in terms of determining its position in a nomological network, we calculated correlations with a theoretically related construct, the TOQS and its subscales^8^. In that respect, we expected all subscales of ACCQ to be positively correlated with ‘Performance worries’, ‘Task-irrelevant thoughts’ and ‘Escaping thoughts’ of the TOQS. In addition, we determined internal reliability using two different measures, namely Cronbach’s alpha and composite reliability. Furthermore, we tested the concurrent validity of the ACCQ through correlations with athletes’ satisfaction in three different domains (i.e., general in life, sporting development and athletic performance in the last season) and athletes’ subjective performance evaluation (i.e., overall performance in the last season, the percentage of peak performance retrieval in their last season, and the percent confidence in achieving their goals in the upcoming season). Regarding the three satisfaction ratings, we expected negative correlations with the ACCQ subscales. The same applies to the correlations between the ACCQ subscales and the athletes’ subjective performance evaluation. Finally, to provide further information for the reader, we conducted population-based differences between the gender, sport-type, and performance level of the athletes.

## Method

### Procedure

Athletes from various team and individual sports throughout United Kingdom and USA were invited to participate in the study via Prolific, a platform for online research participant recruitment. Only athletes who had a coach, were active in organized sports and regularly participate in competitions, were based in the U.K and USA (first language English) were approached to take part (*n* ~ 4.344). Athletes were informed about the nature and the procedure of the study and gave consent before completing the questionnaires. Participation was voluntary and participants received financial compensation. The study was carried out in compliance with the Declaration of Helsinki^16^ and ethical approval was granted from the local Review Board of the Institute of Psychology at the Johannes Gutenberg-University Mainz.

## Participants

We considered an a priori participant: item ratio of 10:1 as a suitable sample size for performing CFA^17,18^. Therefore, we aimed for a minimum sample size of *N* = 260 (26 ACCQ items x 10). Overall, 320 athletes accessed the online survey platform SoSci-Survey^19^ in July 2024, taking on average 8.20 min. However, data from 42 athletes were excluded due to poor data quality (i.e., control items were answered incorrectly).

Thus, the final data set consists of 278 athletes (*M*_age_ = 27.64, *SD* = 8.74, age range = 16–68; female = 78, male = 197, non-binary = 3) from different team (*n* = 181) and individual (*n* = 97) sports. The most frequently represented team sports were soccer (*n* = 82), basketball (*n* = 23) and cricket (*n* = 6) and the most frequently represented individual sports were swimming (*n* = 14), athletics (track and field; *n* = 9) and golf (*n* = 10). On average, athletes practiced 3.39 training sessions (*SD* = 2.00) in their main sport and 3.31 complementary training sessions (such as strength training or jogging; *SD* = 2.47), resulting in an average activity time of 10.92 h of practice per week (*SD* = 7.60). The average sports experience in the respective sport was 11.32 years (*SD* = 7.70). In addition, the athletes participated on average in 14.05 competitions per year (*SD* = 13.04). Moreover, 24 athletes stated that they were on the national team in their sport at the time of the survey or have already been on a national team. Following Swann et al. (2015) 137 athletes can be assigned to the performance status semi-elite athletes, 128 to the performance status competitive-elite and 13 to the performance status successful-elite.

### Measures

#### Adverse competition-related cognitions

For the present study, the original German version of the ACCQ^10^ was translated into English and back translated into German by two independent bilingual speakers of German and English^20^. In addition, for further specification, the items were then commented on by two bilingual athletes. Based on their evaluation, the authors, who also have a background in competitive sports as athletes and/or sports psychologists, discussed the translated items of the ACCQ in a review session and selected the final item wording.

The ACCQ consists of 26 items and captures a series of thoughts that may occur during a competition on six subscales: ‘Athletic comparison’ (5 items; e.g., “I am not good enough.”), ‘Coach devaluation’ (5 items; e.g.,” My coach didn’t do a good enough job preparing me.”), Devaluation of one’s own performance (7 items; e.g.,” That didn’t work the last time either.”), ‘Appreciation by coach and family’ (3 items; e.g., “My parents should be proud of me.”), ‘Inner resistance against competitions’ (3 items; e.g., “I no longer want to participate in competitions.”), and ‘General exhaustion (3 items; e.g., “I can’t go on any longer.”)(*Note*: all items of the questionnaire can be found in the supplement). Response format is a 5-point scale ranging from ‘1’ (*never*) to ‘5’ (*almost always*). For the evaluation, the sum scores of the respective subscales are calculated and the higher the score, the more frequently adverse competition-related cognitions occur in the respective area. To obtain a total ACCQ score (composite score), the values of all items are summed. However, Michel-Kröhler et al.^10^ recommend that the assessment should be made at the factor level and that the total score should only be used for a quick initial overview. Cronbach’s α in the original study was between .72 and .84 for the subscales and .90 for the ACCQ composite score^10^.

## Cognitive interference

The Thought Occurrence Questionnaire Sport (TOQS^8^, consists of 17 items and measures the interference of own thoughts with concentration on three subscales: ‘Performance worries’ (e.g., “That I’m not going to achieve my goal today.”), ‘Task-irrelevant thoughts’ (e.g., “About what I’m going to do later in the day.“), and ‘Thoughts of escape’ (e.g., “That I want to get out of here.”). Athletes responded on a 5-point scale ranging from ‘1’ (*almost never*) to ‘7’ (*almost always*). For the evaluation, the mean values of the respective subscales are calculated and, as with the ACCQ, the higher the value, the more frequently the thoughts occur in the respective area. Cronbach’s α in the original study was 0.78 for ‘Performance worries’, 0.85 for ‘Situation-irrelevant thoughts’, and 0.90 for ‘Thoughts of escape’^8^.

### Satisfaction ratings

We captured athletes’ satisfaction with their life with the short scale Life satisfaction (L-1^21^, , which consists of the following item: “All things considered, how satisfied are you with your life these days?” In addition, we modified this item to capture athletes’ satisfaction with their overall athletic development and their performance in the last season. In detail, satisfaction with athletic development was evaluated with the item “All things considered, how satisfied are you with your sporting development these days?” (L-1 S) according to Michel-Kröhler et al.^10^ and satisfaction with athletic performance with the item “All things considered, how satisfied are you with your sporting performance last season?” (L-1P). Athletes rated all items on an 11-point scale ranging from ‘0’ (*not satisfied at all*) to ‘10’ (*completely satisfied*).

### Subjective performance evaluation

We assessed athletes’ individual performance evaluations in three ways. First, athletes should rate their performance of the last season with the following item: “My performance in the last season was.” (1 = much worse than I expected; 4 = constant; 7 = much better than I expected, cf^22^. Second, the athletes answered two questions on a visual analog scale (VAS), ranging from 0% to 100%, regarding their peak performance last season (“Please indicate on the percentage scale what percentage of your peak performance you think you achieved last season.”) and their confidence to reach their sporting goals next season (“Please indicate on the percentage scale how confident you are that you will achieve your sporting goals in the coming season.”).

### Data analysis

#### Data screening

First, we checked our data for univariate and multivariate normal distribution using Shapiro–Wilk-test for univariate normality and Mardia’s coefficient for multivariate normality (“mvn”- package^23^ . The analyses revealed neither a normal distribution for the individual items nor a multivariate normal distribution of the data (Mardia Kurtosis = 20.08, *p* < .001^24,25^ . Thus, we considered the data suitable for confirmatory factor analysis (CFA) using robust maximum likelihood estimation (MLR), which computes standard errors and model fit indices that are robust in relation to the relative non-normality of observations^26^.

### Confirmatory factor analysis

A CFA was conducted to test the six-factor structure of the ACCQ and to evaluate factor loadings, error variances, and modification indices. We assessed the goodness of model fit with multiple fit indices and reported the χ^2^-test statistic, the Root Mean Square Error of Approximation (RMSEA) and its confidence interval (90% CI), as well as the Standardized Root Mean Square Residual (SRMR), the Comparative Fit Index (CFI), and the Tucker Lewis Index (TLI). RMSEA-values less than 0.08 indicate an acceptable model and less than 0.06 indicate a good model^27^. For the SRMR index, values should be *<* 0.05 for a good fit and *<* 0.10 for an acceptable fit. Regarding CFI und TLI index, values *>* 0.90 are indicative of a good model fit^27^. In addition, we calculated the Akaike Information Criterion (AIC) and the Expected Cross-Validation Index (ECVI) to compare the tested models. Since both fit indices do not have a specific range of acceptable values, we assumed that the lower the value, the more likely it is that the model can be replicated in other samples.

Following Michel-Kröhler et al.^10^, we also conducted a higher-order model in which the six first-order factors were designed to load onto a second-order factor, namely an overall adverse competitive cognition factor. Marsh^28^ suggests that a hierarchical model is supported if the fit of a higher-order model closely matches that of the corresponding first-order model. However, it is also recommended to specify the Average Extracted Variance (AVE^29^, alongside the standard model fit indices. An AVE value greater than 0.50 indicates that the higher-order factor can sufficiently explain variance in the lower-order factors. Additionally, loadings on the higher-order factor should exceed 0.70 (see also^30^.

### Correlational analyses

We conducted three independent Pearson correlation analysis to examine (1) the inter-correlations of the ACCQ subscales, (2) the construct validity of the ACCQ in terms of a nomological network with the subscales of the TOQS^8^ to measure associations with athletes’ cognitive interference, and (3) the concurrent validity of the ACCQ and satisfaction in three different domains and athletes’ subjective performance evaluation. We corrected *p*-values for multiple comparisons using Holm’s method for each analysis. The following criteria apply to evaluate the correlation coefficients: very high (≥ 0.90), high (≥ 0.70), moderate (≥ 0.50), and low (< 0.50)^31^.

### Reliability analysis

We computed Cronbach’s alpha (α) and the composite reliability (ρ) as measures of internal consistency. According to Nunnally and Bernstein^32^, coefficients of composite reliability and Cronbach’s alpha greater than 0.70 indicate good reliability of test scores (see also^33^.

### Population-based differences

First, we correlated subscales of the ACCQ with participants’ age. In addition, we divided our sample into three performance levels (semi-elite vs. competitive-elite vs. successful-elite; see^34^ and two different sport-types (team vs. individual athletes). Regarding the assignment of performance levels, we must mention that we have categorized the performance levels based on the British sports system and applied them to the athletes from the USA, so that minimal deviations may have occurred there. Second, we conducted a three-way MANOVA with performance level, sport-type, and gender to examine differences in the six subscales of adverse competition-related cognitions. However, due to small sample sizes, we had to exclude athletes who identify as binary (*n* = 3) in gender category and successful-elite athletes (*n* = 13) in performance level category from the analysis. Beforehand, we tested the assumption of independent observations with intercorrelations of the dependent variables as well as multivariate normality with the Shapiro–Wilk-test (*p* > .05) and univariate normality with the Kolmogorov-Smirnov-Test^35^. In addition, we evaluated the equality of variance–covariance matrices with Box’s M test (*p* > .05). Results indicated neither a multivariate normal distribution (W = 0.931. *p* < .001) nor a normal distribution for the six subscales of the ACCQ (all *p*’s < 0.001, devaluation of one’s own performance: *p* < .01). Moreover, the Box M results showed that both genders, sport-types, and performance levels, have similar variance-covariance matrices (*p* > .05). In general, we reported partial eta squared (ηp²) as corresponding effect size with the following criteria for small, medium, and large effect: 0.01, 0.06, and > 0.14^36^.

## Results

### Construct validity in terms of factorial validity

**Fig. 1:**
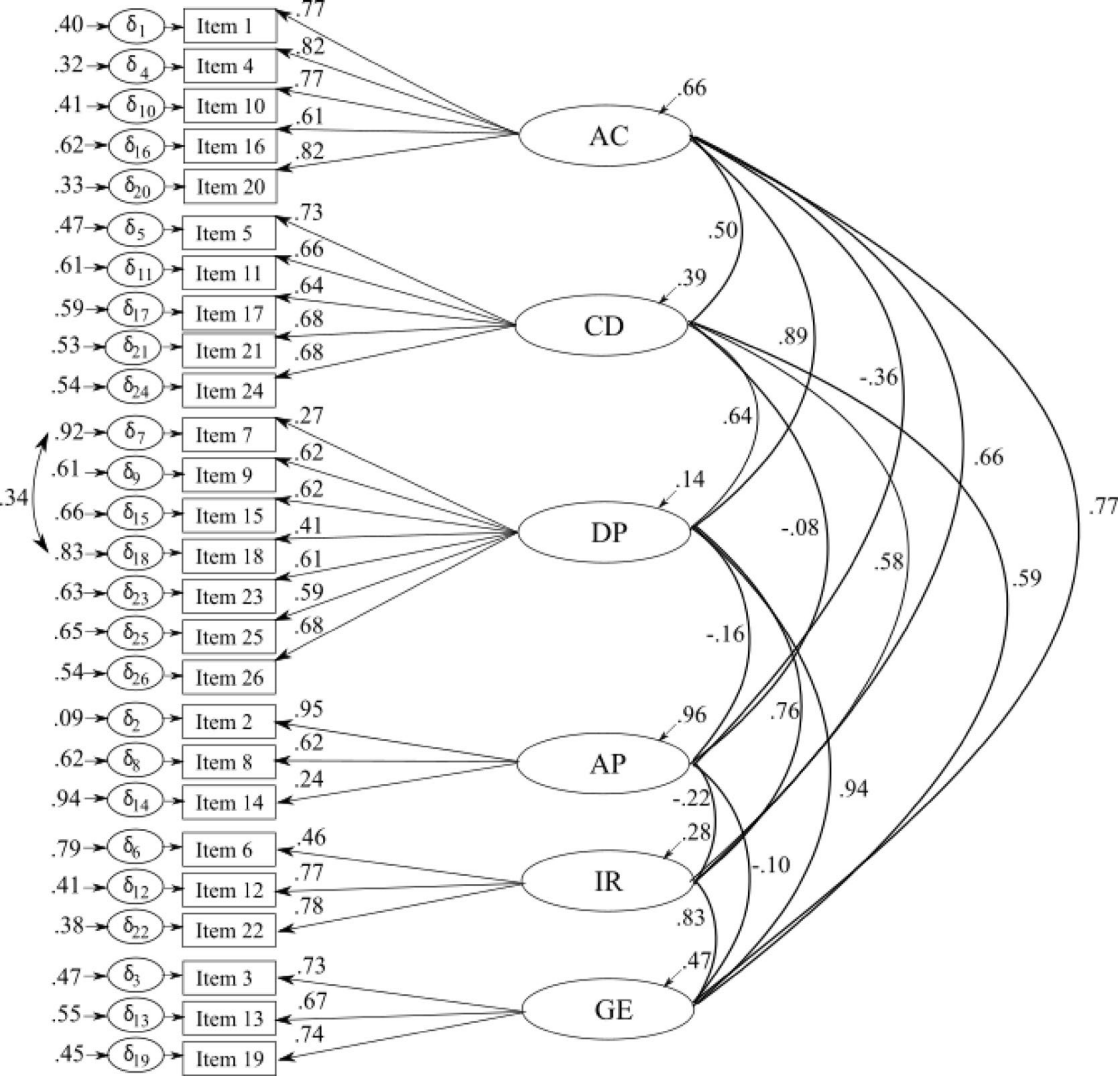
6-factor 26-item solution of the English version of the ACCQ with standardized factor loadings and error variances. AC = Factor ‘Athletic’ comparison, CD = Factor ‘Coach devaluation’, DP = Factor ‘Devaluation of one’s own performance’, AP = Factor ‘Appreciation by coach and family’, IR = Factor ‘Inner Resistance against competitions’, GE = Factor ‘General exhaustion’.

Results of the initial CFA revealed only a partial acceptable fit to the expected six-factor solution, *N* = 278, *χ*²(284) = 585.268, *p* < .001, CFI = .885, TLI = .869, RMSEA = .065[.058, .073], SRMR = .078, AIC = 18271.591, ECVI = 2.816. Therefore, we decided to perform a second CFA in which we considered the proposed modification in the form of a covariation of the residuals of Item 7 (“I am not allowed to fail.”) and Item 18 („I can’t allow myself to make mistakes.”). The result of the second CFA showed a better model fit within the recommended ranges compared to the first model: *N* = 278, *χ*²(284) = 506.011, *p* < .001, CFI = 0.915, TLI = 0.903, RMSEA = 0.056[0.048, 0.064], SRMR = 0.074, AIC = 18184.943, ECVI = 2.504. Standardized factor loadings ranged from 0.406 to 0.954 except for Item 7 and Item 14 (“I don’t want to disappoint my coach”), which had factor loadings of 0.275 and 0.244. Error variances were between 0.324 and 0.940 (for more details see Fig. 1).

A slightly worse but still comparable model fit was achieved with the higher-order model –in which the six first-order factors were designed to load onto a second-order factor, namely an overall adverse competitive cognition factor: *N* = 278, *χ*²(292) = 548.855, *p* < .001, CFI = 0.902, TLI = 0.891, RMSEA = 0.059[0.052, 0.067], SRMR = 0.078, AIC = 18215.484, ECVI = 2.614. However, the AVE was *>* 0.50 only for two factors (‘Athletic comparison’ and ‘General exhaustion’). In addition, except for two factors (‘Coach devaluation’: λ = 0.634 and ‘Appreciation by coach and family’: λ = − 0.229), the loadings on the higher-order factor reached the recommended value of > 0.70. For details on the standardized factor loadings and the error variances, see Figure S1 in the Supplement.

### Construct validity in terms of a Nomological network

Descriptive statistics and correlations are summarized in Table 1. As expected, subscales of the ACCQ (except for ‘Appreciation by coach and family’) were significant and positively correlated with the three subscales of the TOQS. The stronger the adverse competition-related cognitions in the different subscales the stronger the athletes’ performance worries, task-irrelevant and escaping thoughts.

**Table 1:** Mean (M), standard deviation (SD), internal reliability coefficients (α,* ρ)*, inter-correlations for the ACCQ subscales as well as correlations of all study variables used separated by three different analyses.

		M (SD)	α/ρ	1	2	3	4	5	6	7
Construct validity in terms of Inter-correlations of the ACCQ subscales										
1	Athletic comparison	11.68 (4.21)	0.87/0.87							
2	Coach devaluation	9.21 (3.24)	0.81/0.81	0.41***						
3	Devaluation of one’s own performance	17.44 (5.12)	0.77/0.77	0.64***	0.45***					
4	Appreciation by coach and family	10.33 (2.61)	0.60/0.66	− 0.14	− 0.04	0.16*				
5	Inner resistance against competitions	5.60 (2.39)	0.69/0.72	0.52***	0.42***	0.48***	− 0.08			
6	General exhaustion	5.71 (2.36)	0.76/0.76	0.61***	0.46***	0.66***	< 0.01	0.61***		
7	Composite score (ACCQ)	59.97 (14.03)	0.90/0.90							
Construct validity in terms of nomological network										
8	Performance worry (TOQS)	3.34 (1.23)	0.76/0.79	0.61***	0.45***	0.58***	0.05	0.39***	0.48***	0.65***
9	Task-irrelevant thoughts (TOQS)	2.78 (1.38)	0.88/0.88	0.27***	0.29***	0.37***	0.05	0.36***	0.34***	0.41***
10	Escaping thoughts (TOQS)	2.37 (1.26)	0.92/0.92	0.54***	0.52***	0.61***	< 0.01	0.61***	0.66***	0.72***
Concurrent validity of the ACCQ										
11	Satisfaction general (L-1)	6.88 (1.97)		− 0.31***	− 0.23**	− 0.22**	0.14	− 0.21**	− 0.24***	− 0.28***
12	Satisfaction sporting development (L-1 S)	6.42 (2.04)		− 0.41***	− 0.33***	− 0.25***	0.21**	− 0.29***	− 0.29***	− 0.35***
13	Satisfaction performance	6.27 (2.06)		− 0.46***	− 0.38***	− 0.33***	0.17*	− 0.31***	− 0.34***	− 0.43***
14	Performance last season	4.60 (1.09)		− 0.42***	− 0.32***	− 0.19*	0.20**	− 0.29***	− 0.25***	− 0.32***
15	Peak performance last season (%)	65.89 (18.09)		− 0.38***	− 0.32***	− 0.22**	0.11	− 0.27***	− 0.27***	− 0.34***
16	Confidence goal achievement coming season (%)	69.21 (21.86)		− 0.44***	− 0.29***	− 0.18*	0.27***	− 0.36***	− 0.31***	− 0.33***

*p < .05, **p < .01, ***p < .001, α = Cronbach’s alpha, ρ = composite reliability. Internal reliability team version of the TOQS: performance worry: α = 0.85/ ρ = 0.85; Task-irrelevant thoughts: α = 0.90/ ρ = 0.90; Escaping thoughts: α = 0.93/ ρ = 0.93.

### Scale reliability

We calculated Cronbach’s alpha coefficients as well as composite reliability as measures of internal consistency of the ACCQ and its subscales. Table 1 shows that with a few exceptions, alpha coefficients as well as composite reliability coefficients were in a good range α > 0.70^32^. With α < 0.70, two subscales (i.e., ‘Appreciation by coach and family’ and ‘Inner resistance against competitions’) were not ‘good’, but still in an acceptable range^32^.

### Concurrent validity of the ACCQ

Pearson’s correlation coefficients indicated significant low and negative correlations between all subscales of the ACCQ (except for ‘Appreciation by coach and family’) and satisfaction with life as well as satisfaction with one’s own athletic development and athletic performance. According to this, the satisfaction of athletes in all three domains is higher, the lower their adverse competition-related cognitions. Interestingly, correlation between the subscale ‘Appreciation by coach and family’ and all three satisfaction domains were significant low (except for general satisfaction with life) and positive, meaning that the higher the perceived appreciation by coach and family, the higher the athletes’ satisfaction in all three areas.

Regarding the subjective performance evaluation of the athletes’ a similar pattern of results appeared. There were significant low but negative correlations with five of six ACCQ subscales. The exception is again the subscale ‘Appreciation by coach and family’, which showed significant low but positive associations with athletes’ evaluated performance in the last season and the confidence of achieving their goals in the coming season. The correlation with the rated retrieval of peak performance in the last season was not significant.

### Population-based differences

Tables 2, 3 and 4 provide Pearson correlation coefficients for athletes’ age as well as descriptive values (summed mean values and standard deviations) for men and woman, for individual and team athletes, as well as for semi-elite and competitive-elite athletes for the six subscales of the ACCQ. We conducted a three-way MANOVA with gender, performance level, and sport-type as independent variables to test whether potential effects might be nested across groups.

**Table 2:** Effect size differences between gender and bivariate correlations with participant age for the English version of the ACCQ.

	Age	Gender											
		Female (*n* = 78)				Male (*n* = 197)				Non-binary (*n* = 3)			
	*r*	*M*	*SD*	*Md*	*95%CI*	*M*	*SD*	*Md*	*95%CI*	*M*	*SD*	*Md*	*95%CI*
Athletic comparison	0.05	12.09	4.62	11.00	[11.04, 13.13]	11.55	4.05	11.00	[10.98, 12.12]	9.67	3.05	9.00	[2.08, 17.25]
Coach devaluation	0.03	9.10	3.11	9.00	[8.40, 9.80]	9.29	3.30	9.00	[8.82, 9.75]	6.67	0.58	7.00	[5.23, 8.10]
Devaluation of one’s own performance	− 0.06	17.63	5.76	16.50	[16.33, 18.93]	17.39	4.87	18.00	[16.70, 18.07]	15.67	3.75	14.00	[6.26, 25.07]
Appreciation by family and coach	− 0.16	10.74	2.64	11.00	[10.20, 11.28]	10.18	2.64	10.00	[9.81, 10.55]	10.00	5.29	12.00	[-3.14, 23.14]
Inner resistance against competitions	0.03	6.40	2.70	6.00	[5.79, 7.01]	5.31	2.20	5.00	[5.00, 5.62]	4.00	0.00	4.00	-
General exhaustion	0.02	6.05	2.34	6.00	[5.52, 6.58]	5.59	3.37	5.00	[5.26, 5.92]	4.33	2.31	3.00	[-1.40, 10.07]
ACCQ composite score	− 0.02	62.01	15.38	63.00	[58.54, 65.48]	59.31	13.43	58.00	[57.42, 61.20]	50.33	12.22	53.00	[19.98, 80.69]

*p < .05, **p < .01, ***p < .001, *r* = Pearson correlation coefficient, *M* = mean values of respective sum scores, *SD* = standard deviation, *Md* = median, *CI = confidence interval.*

**Table 3:** Effect size differences between individual and team athletes for the E nglish version of the ACCQ.

	Individual athletes (*n* = 97)				Team athletes (*n* = 181)			
	M	SD	Md	95%CI	M	SD	Md	95%CI
Athletic comparison	12.03	4.08	12.00	[11.21, 12.85]	11.49	4.28	11.00	[10.86, 12.12]
Coach devaluation	8.74	3.14	8.00	[8.11, 9.37]	9.46	3.27	10.00	[8.98, 9.94]
Devaluation of one’s own performance	17.25	5.00	17.00	[16.24, 18.25]	17.54	5.19	17.00	[16.78, 18.30]
Appreciation by family and coach	10.21	2.69	10.00	[9.66, 10.75]	10.40	2.57	10.00	[10.03, 10.78]
Inner resistance against competitions	5.99	2.32	6.00	[5.52, 6.46]	5.40	2.41	5.00	[5.04, 5.75]
General exhaustion	5.65	2.33	5.00	[5.18, 6.12]	5.73	2.39	5.00	[5.38, 6.08]
ACCQ Composite	59.86	13.70	60.00	[57.10, 62.63]	60.03	14.25	59.00	[57.94, 62.12]

*M* = mean values of respective sum scores, *SD* = standard deviation, *Md* = median, *CI = confidence interval.*

**Table 4:** Effect size differences between semi-elite, competitive-elite and successful-elite athletes for the english version of the ACCQ.

	Semi-elite athletes (*n* = 137)				Competitive-elite athletes (*n* = 128)				Successful-elite athletes (*n* = 13)			
	M	SD	Md	95%CI	M	SD	Md	95%CI	M	SD	Md	95%CI
Athletic comparison	12.48	3.86	13.00	[11.83, 13.13]	11.11	4.48	10.00	[10.32, 11.89]	8.85	2.85	9.00	[7.12, 10.57]
Coach devaluation	9.30	3.30	9.00	[8.74, 9.86]	9.24	3.25	9.00	[8.67, 9.81]	7.92	2.25	8.00	[6.56, 9.28]
Devaluation of one’s own performance	17.95	4.81	18.00	[17.14, 18.76]	16.89	5.52	16.00	[15.92, 17.86]	17.46	3.64	17.00	[15.26, 19.66]
Appreciation by family and coach	10.05	2.49	10.00	[9.63, 10.47]	10.42	2.71	10.00	[9.95, 10.89]	12.46	1.81	13.00	[11.37, 13.55]
Inner resistance against competitions	5.77	2.22	5.00	[5.40, 6.15]	5.43	2.62	5.00	[4.97, 5.89]	5.54	1.71	6.00	[4.50, 6.57]
General exhaustion	5.93	2.31	6.00	[5.53, 6.32]	5.52	2.43	5.00	[5.10, 5.95]	5.15	2.11	4.00	[3.87, 6.43]
ACCQ Composite	61.48	12.52	62.00	[59.36, 63.60]	58.62	15.70	56.00	[55.87, 61.63]	57.38	10.30	58.00	[51.16, 63.61]

*M* = mean values of respective sum scores, *SD* = standard deviation, *Md* = median, *CI = confidence interval.*

The first analysis showed no significant correlations between athletes’ age and all subscales of the ACCQ. Furthermore, the three-way MANOVA indicated no significant main effects for gender: λ = 0.970, *F*(6, 249) = 1.25, *p* = .282, ηp² = 0.029 and sport-type, λ = 0.974, *F*(6, 249) = 1.09, *p* = .367, ηp² = 0.025. In contrast, there was a significant main effect for performance level, λ = 0.955, *F*(6, 249) = 2.17, *p* = .046, ηp² = 0.049. A significant between-subject effect was revealed for ‘Athletic comparison’, *F*(1,260) = 6.93, *p* = .009, ηp² = 0.026, and ‘Devaluation of one’s own performance’, *F*(1,260) = 2.92, *p* = .089, ηp² = 0.011, indicating both times higher values for semi-elite athletes compared to competitive-elite athletes. Furthermore, there were no significant interaction effects between gender, sport-type, and performance level: gender x sport-type: λ = 0.954, *F*(6, 249) = 1.96, *p* = .071, ηp² = 0.045, gender x performance level: λ = 0.975, *F*(6, 249) = 1.07, *p* = .382, ηp² = 0.025, sport-type x performance level: λ = 0.970, *F*(6, 249) = 1.27, *p* = .272, ηp² = 0.030, gender x sport-type x performance level: λ = 0.969, *F*(6, 249) = 1.33, *p* = .245, ηp² = 0.031.

## Discussion

The present study aimed at confirming the 6-factor structure of the ACCQ through confirmatory factor analysis in a sample of English-speaking athletes. In addition, we took further steps to assess the reliability and validity of the English version of the ACCQ, by (1) determining the convergent and divergent validity of the ACCQ by application of a nomological network with theoretically related constructs, (2) determining internal consistencies of the subscales and total score using two measures (Cronbach’s α & composite reliability) and, (3) examining the concurrent validity with correlations between the ACCQ and its subscales and athletes’ satisfaction in three different domains and their subjective performance evaluation. Lastly, we calculated population-based differences in relation to gender, sport-type and performance level, to provide the reader with additional information about the ACCQ.

Regarding the English translation of the ACCQ, results of a first CFA showed a somewhat unsatisfying model fit of the data. A closer look at the model revealed an extremely high modification index of Item 7 and Item 18. Both items relate to cognitions about failures and potential consequences, which is part of the ‘Devaluation of one’s own performance’ subscale. Thus, we decided to conduct a second CFA considering the covariation of the residuals of these items. In addition, Item 18 was also conspicuous in the German validation study^10^ due to its covariation with another failure-related item. Moreover, Item 7 and Item 14 showed a very low factor loading (< 0.30). Regarding Item 14, this could be because the wording of the item differs from that of the other two items. However, to be able to carry out cross-cultural comparisons across different samples in the future, we decided to retain the two items and not to make any further adjustments to the ACCQ. Nevertheless, future studies should examine whether this is a general structural problem or an actual content-related problem (considering also other, more balanced samples; see Limitations).

However, what seems most surprising compared to the German version of the ACCQ is that the factor ‘Appreciation by coach and family’ is negatively related to the other factors, so that the related cognitions appear to be perceived comparatively positively in the sample of English-speaking athletes. This could perhaps be because the formulations “should be” are not understood as a rigid assertion of desires and demands that certain conditions must or must not exist^37,38^, but much more as motivation to achieve a good performance. Future research should therefore examine the relationships with rational and irrational beliefs (e.g., with the irrational Performance Beliefs Inventory^9^, to finally determine whether these cognitions are perceived as negative and thus potentially performance-limiting or positive and performance-enhancing. Another reason might be the performance level of the athletes. It can be assumed that the desire for recognition from the coach and family increases as the level of performance rises, as the athletes may feel that they can give something back to the coaches and family who invest a lot of time (and money) and support as well as encourage them for years through their successes or achievements. However, we cannot investigate this sufficiently in our sample, as it contains only a few athletes who are active at successful-elite level and none above.

With regard to the higher-order factor model, results showed a slightly different but comparable fit. However, the average extracted variance of only two from six factors was above the recommended value of 0.50, and loadings on the higher-order factor did not all exceed the recommended value of 0.70. It must therefore be assumed that additional information for predicting important outcomes may be lost if all manifest variables are simply aggregated to a higher-order ACCQ score^30^.

Finally, the construct validity of the ACCQ was demonstrated by significant and positive correlations with cognitive interference measured by the TOQS, and internal reliability was confirmed by satisfactory Cronbach’s alpha and composite reliability coefficients.

Regarding the concurrent validity of the ACCQ, the relationships with athletes’ satisfaction in three different domains (i.e., life, sporting development, and athletic performance) were significant and negative as expected, suggesting that the more negative competition-related cognitions athletes experience, the less satisfied they are. Of particular interest here is that the adverse competition-related cognitions are related to the athletes’ general life satisfaction, which is in line with the results of Michel-Kröhler et al.^10^. Moreover, this relationship could be an indicator of the importance of sport in their lives^39^. Athletes who have a high athletic identity can attach great personal importance to their sport compared to other things in life, so that the experiences, emotions, and cognitions associated with sport are so inherent that they are transferred to their general life satisfaction. This is consistent with Poucher and Tamminen^40^, who found that athletes with a high athletic identity may have difficulty adjusting in other areas of life if they focused solely on athletic goals, which would explain the carryover to general dissatisfaction. However, to confirm this assumption, future studies should also measure athletic identity^41^ in addition to ACCQ and satisfaction.

The second correlation analysis with the subjective performance evaluation showed a similar pattern of results, indicating the more adverse competition-related cognitions athletes experience the more negative their performance evaluation and the lower athletes’ confidence in achieving their goals next season. Not surprisingly, the correlations between the three subjective performance ratings were each most strongly correlated with the ACCQ subscale ‘Athletic comparison’, which contains items relating to one’s own performance evaluation (e.g., “I can’t perform well.”) in addition to the items relating to sporting comparison with others. Finally, when interpreting the correlations related to the concurrent validity of the ACCQ it should be noted that this does not apply to the ‘Appreciation by coach and family’ subscale. The negative correlations with the other ACCQ subscales are also evident in the inverse correlations with satisfaction and subjective performance evaluation.

Regarding the population-related differences, the results are somewhat more difficult to interpret, as no findings from other studies are yet available. However, if one compares the results with the findings of related constructs such as sport-specific rumination^42^, irrational performance beliefs^43,9^ or cognitive interference^44^, they pointed all in the same direction regarding athletes’ age and gender. With increasing age, thoughts and beliefs decrease, and female athletes show higher values compared to male athletes^42,43,9^. Regarding the performance level and the sport-type, different patterns of results emerged. While in one study no differences were found between the performance levels^43^, another study showed differences between amateur and semi-professional athletes^9^. Furthermore, Lane et al.^44^ reported higher cognitive interference in individual athletes compared to team athletes, while Michel-Kröhler and Turner^43^ found in two dimensions of irrational beliefs higher values for team athletes. Furthermore, our study showed neither a correlation with age nor differences between the genders, which would contradict previous findings from the aforementioned studies. In contrast, there was a difference between the performance levels in our study, indicating higher values in ‘Athletic comparison’ and ‘Devaluation of one’s own performance’ for semi-elite athletes compared to competitive athletes, which fits to the findings that athletes with lower performance level showed higher values in certain dimensions of irrational performance beliefs compared to athletes with a higher performance level^9^.

### Limitations and future research

Some potential shortcomings must be addressed before using the ACCQ. First, our sample is a convenience sample that was collected using the Prolific platform. Recruiting via this platform has some advantages, such as fast data collection for a selected target group; however, it also has some disadvantages. One drawback is a self-selection bias in the sense that rapid responders (first-come, first-served participation), athletes interested in the subject of the study and athletes of a higher socio-economic status are overrepresented. To better generalize the results to a broader range of athletes, future studies should combine survey methods to increase opportunities for equal participation and include individuals from different socioeconomic backgrounds.

In addition, the generalizability of the results is also limited as the current sample consists of twice as many men as women, as well as twice as many team athletes as individual athletes. Therefore, future studies should examine differences again in more balanced groups regarding gender and sport-type. The same applies to the performance level. Our sample included only thirteen athletes of the successful-elite performance level and none from a level above, so that analyses were limited to two performance levels.

Furthermore, we have categorized the performance levels based on the British sports system and applied them to the athletes from the USA. This may pose a problem and, therefore, the results should be interpreted with caution. However, this only affects one of five categories that are used to determine the performance level according to Swann et al.^34^, namely the category ‘competitiveness of the sport in the athlete’s country.’ Here the sports are rated according to ranks in country (top 5 or top 5–10), with a 50% overlap in the top 10 sports between the UK and the USA (i.e., soccer, tennis, golf, boxing and motor sport).

In addition, our sample only includes athletes aged 18 and older and have a high mean age compared to the general peak performance age. According to Michel-Kröhler et al. [42, see also 44], adverse competition-related cognitions seem to be also very relevant in adolescent. The characteristics of this age group are the junior-to-senior transition level. It is likely that this phase is accompanied by increased levels of adverse competition-related cognitions that can more easily interfere with performance. Therefore, future research should examine competition-related cognitions profiles of adolescent athletes longitudinally as they progress through different athletic transitions from junior to senior status. In addition, further studies are needed to evaluate whether the ACCQ provides an assessment in more specific age groups.

To summarize, to obtain more information for appropriate applications, future studies should examine differences in adverse competition-related cognitions across age, gender, performance level, sport-type in balanced samples and consider measurement invariance for these aspects.

### Practical application

With its six factor structure, the ACCQ is suitable for an initial “screening” that provides a first overview of the frequency of adverse thoughts that occur immediately before or during a competition. A detailed analyses, for instance, by creating individual profiles that reflect the athletes’ characteristics on the various dimensions, can be used to apply targeted intervention and prevention measures. In addition, changes in athletes’ mindset over the course of the sporting season can be identified through the application of the ACCQ. This could enable sports psychologists and coaches to work more specifically with athletes in certain areas in the future.

To investigate the direct link between adverse thoughts and performance, the ACCQ could in future also be used immediately before and after a competition to identify thoughts that are directly related to the athletes’ performance or behavior. For this purpose, however, it would be advisable to reduce the number of items in the ACCQ and adapt the instructions to capture the current state (for further implications for research and applied sports psychology, see^10^.

## Conclusion

In conclusion, results provide confirmatory support to the notion that the ACCQ shows factorial validity for application in English-speaking samples of athletes. Compared to existing questionnaires in the same research area, the ACCQ allows sport-specific adverse competition-related thoughts to be measured at their core. More specifically, the application of the ACCQ enables the identification of different areas (i.e., athletic comparison, coach devaluation, devaluation of one’s own performance, appreciation by coach and family, inner resistance against competitions, and general exhaustion) that can potentially have an impact on sport performance or athlete well-being. Thus, ACCQ is a useful and valid measure for assessing various negative competition-related cognitions that offers a wide range of potential applications in research and sport psychology practice.

## Data Availability

The data that support the findings of this study are openly available in OSF at: https://osf.io/m5kr7/?view_only=e743d99bfaea430f8859955bef1bf182.
