# Correction: Adverse competition-related cognitions and it’s relation to satisfaction and subjective performance: a validation study in a sample of English-speaking athletes

**DOI:** 10.1038/s41598-025-24763-5

**Published:** 2025-10-29

**Authors:** Alena Michel-Kröhler, Michèle Wessa, Stefan Berti

**Affiliations:** 1 Department of Clinical Psychology and Neuropsychology, Institute for Psychology, Johannes Gutenberg-University Mainz, Wallstrasse 3, 55122 Mainz, Germany; 2 Department of Neuropsychology and Psychological Resilience Research, Central Institute of Mental Health, Mannheim, Germany; 3 German Cancer Research Center (DKFZ), Division of Cancer Survivorship and Psychological Resilience, Heidelberg, Germany; 4 Leibniz Institute for Resilience Research (LIR), Mainz, Germany

Correction to: *Scientific Reports* 10.1038/s41598-025-16077-3, published online 25 August 2025

The original version of this Article contained errors in the name of authors Alena Michel-Kröhler, Michèle Wessa and Stefan Berti, which were incorrectly given as Michel-Kröhler Alena, Wessa Michèle and Berti Stefan.

The original Article has been corrected.

